# Improving the Jump Shots of U12 Junior Basketball Players by Implementing a Combined Program of Plyometric and Coordination Exercises Using MyVert Technology

**DOI:** 10.3390/s24123993

**Published:** 2024-06-20

**Authors:** Antonia Radu, Dana Badau, Adela Badau

**Affiliations:** 1 Faculty of Physical Education and Mountain Sports, Transilvania University, 500068 Brasov, Romania; 2 Department of Environmental Sciences, Physics, Physical Education and Sport, “Lucian Blaga” University of Sibiu, 550012 Sibiu, Romania

**Keywords:** movement analysis, smart sensors, technology, plyometric, coordination exercises, basketball, jump shots

## Abstract

The aim of this study was to investigate the impact of the implementation of an experimental program with combined plyometric and coordination exercises for a time interval of 6 months aimed at improving the jump shots of U12 junior players through the use of information technologies. One hundred seventeen female basketball players, aged between 10 and 12 years (U12), participated in this study. The study subjects were divided into two groups: the experimental group (EG), with 60 (51.3%) subjects, and the control group (CG), with 57 subjects (48.7%). The 6-month experiment program implemented in the experimental group included exercises that combined coordination exercises with plyometric exercises in the execution of throwing skills and skills specific to the basketball game by using the MyVert portable smart sensor. This study included an initial test and a final test, in which three motor tests adapted to the specifics of the basketball game were applied in order to evaluate jump shots: a throw-after-step test, a standing shot test and a shot-after-dribbling test. Only the results of the experimental group showed statistically significant progress (*p* < 0.05) between the final and initial testing in all three motor tests for the following parameters: maximum jump height (cm), average jump height (cm), power (watts/kg) and successful shots (no). The gains of the control group were not statistically significant in any test. It should be noted that the number of throws scored in the basket of the experimental group increased significantly, a fact highlighted by the very large size of Cohen’s value > 3 in all the tests of this study. The results of the experimental group as a result of the implementation of the experimental training program using MyVert technology were superior to the results of the control group. The practical implications of the present study will contribute to the optimization of the athletes’ training methodology in order to improve the physical and technical levels in relation to the peculiarities of age and training level.

## 1. Introduction

Basketball is a team sports game characterized by complexity and technicality, and it has a continuous dynamic in terms of physical and technical training in correlation with the performance objectives specific to each level of training. The interrelationship between the technical level and the physical capacity of the players supports the optimization of the performance level of the athletes [1,2]. The use of portable sensor technology in basketball allows the scientific quantification and monitoring of training and sports performance in terms of physical and technical parameters [3,4,5].

The progress of science and technology has facilitated the diversification of portable sensor technology that, through the data provided, contributes to optimizing the needs of athletes and coaches [5,6]. The benefits of technology are undeniable in any field of activity, and sports represents a field in which the innovative implications of technology are involved in continuous diversification and specialization with a major impact on future sports performances [7,8]. The contribution of technology in sports is decisive in the monitoring and evaluation of sports performance parameters. The specialization of these technologies facilitates the quantification of the main physical and technical parameters in relation to the characteristics and specifics of different sports. The use of technology in the sports training process allows the collection of data and feedback in real time, which can contribute to the optimization of training, especially in the initiation and junior stage specific to basketball [9,10,11]. In the last decade, numerous research studies have focused on studying the impact of the use of different informational technologies and portable smart sensors in basketball, aiming at improving the sports performance and the physical and technical parameters of the athletes [12,13,14]. The use of information technologies allows for the identification of positive aspects and aspects that lead to the disadvantage of athletes through execution errors or the insufficient development of some physical parameters [15,16]. The correction and modeling of basketball training requires a permanent update to take into account the particularities of the athletes, the sports experience and the training and performance objectives [17,18].

The level of technique and the level of physical fitness combined with sports experience are major components of ensuring success in basketball. The efficiency of basketball throws is conditioned by a series of parameters like coordination capacity and physical fitness, including hand–eye coordination, handball, the parameters of the vertical jump when making shots at the basket, the accuracy of the shots, etc. [19,20]. During the initiation time in basketball, a major objective is the correct acquisition of execution techniques in order to make executions more efficient and ensure an optimal level of physical fitness [21,22]. Coordination in making basketball shots requires intersegmental control in conditions of movement, precision, spatial orientation, adversity, etc. [23,24]. Coordination abilities are essential components in the process of learning and perfecting technical skills and are made up of the following components: general coordination, rhythm, spatial orientation, precision, the ability to combine movements and reaction time [2,25,26,27]. In the game of basketball, the efficiency of the throws is conditioned by the context of the game (throwing in adverse conditions, throwing after moving, throwing in jumping conditions, throwing from different distances to the basket, etc.) and the training level of the players [28,29,30]. The efficiency of the jump shots is conditioned by the height of the jump, the impulse power of the lower limbs, the posture and body alignment during the flight, the execution technique, the hand–eye coordination and the accuracy of the throw [31,32,33]. Plyometric exercise programs have proven their effectiveness in improving players’ ability to perform various technical skills specific to basketball [34,35]. In sports training, all these aspects require special attention from coaches and athletes in order to maximize the physical and technical potential specific to basketball.

Studies on the efficiency of shots, jumps and jump shots in game-specific conditions, such as those preceded by movement or from standing or preceded by other technical actions, have been the target of numerous research studies [36,37]. The results of these studies highlight the importance of individualizing and adapting training according to characteristics like age and level of sports experience [38,39,40]. Studies that identify how the combination of plyometric exercises and coordination exercises adapted to the specifics of the basketball game contribute to the improvement of jump shots in training conditions are very few in number and do not cover the aspects of the present study. We consider the application of a 6-month experimental program combined with plyometric and coordination exercises in order to improve the characteristics of jump shots in conditions close to those in the game. The experimental program was adapted to the particularities of age (10–12 years) and the level of sports training (juniors U12, sports experience of at least 2 years), which will contribute to the optimization of sports performance in basketball.

Basketball combines the skills of running and jumping with those of passing, dribbling and throwing, which are based on a high level of strength, coordination, speed and endurance. The specifics of the basketball game include the ability to perform the skill of throwing the basketball in conditions of lateral movement, from the jump, pirouette, etc. The success and efficiency of basketball hoop shots are conditioned by the coordination of the segments when making shots from the spot or from the jump, and the jump shot is influenced by the parameters of the jump (height and power of the jump). Most coaches approach the sports training of junior players by carrying out separate programs to improve the jump parameters, respectively, for coordination and implicit technique. The experimental program implemented in the present study was adapted in such a way as to increase the efficiency of the training by designing and practicing exercises that combine plyometrics with coordination in technical conditions specific to basketball. We consider that the novelty of our study consists of the design and implementation of a combined program of plyometric and coordination exercises that uses the MyVert sensor [41] in order to improve the technical level of the players. This study focuses on jump shots performed in conditions adapted to the game of basketball (shots were combined and preceded by other technical actions). We also designed and applied three tests to evaluate the jump parameters, power and effectiveness of the jump shots, which were adapted to the specifics of the basketball game (the jump shot was preceded by various technical actions).

The aim of this study was to investigate the impact of the implementation of an experimental program of combined plyometric and coordination exercises for a time interval of 6 months, which aimed at improving the jump shots of junior U12 players in game-specific conditions (jump shots were preceded by various movements and technical actions).

## 2. Materials and Methods

### 2.1. The Design of this Study

The research was carried out between April and November 2023, staged in an initial testing session (IT) (the first week of this study) and a final testing session (FT) (the last week of this study) and the implementation time interval of a combined experimental program of plyometric and coordination exercises. The training program had a duration of 6 months, including 24 weeks. The experimental program was implemented in a total of 48 trainings organized in 2 trainings/week/90 min. It should be mentioned that during this study, the sportswomen of this study, both from the experimental and the control group, performed an average of 4 training sessions per week, but of these, the experimental group performed 2 training sessions per week according to the experimental program. The experimental program for the experimental group included exercises that combined coordination exercises with plyometric exercises in the execution of throwing skills and skills specific to the basketball game by using the MyVert portable smart sensor [41]. The training program had a duration of 6 months, including 24 weeks. The exercises included in the experimental program were structured according to their specifics as follows: for coordination, 22 exercises were applied (coded C1–C22); for plyometrics, 29 exercises were practiced (coded P1–P29); and there were 15 combined coordination–plyometric exercises (coded C-P1-C-P15). During this study, the control group undertook a physical and technical training session aimed at improving basketball shots through specific exercises without the use of smart technologies or sensors (Table 1).

The tests for evaluating the level of coordination were applied under the same conditions and at the same time for both groups. The order of the tests was as follows: the shot-after-step test, the standing shot test and the shot-after-dribbling test. Each test was performed 2 times, and the best result recorded by each athlete was taken into account for this study. This study was made while respecting the principles of the Helsinki Declaration; all subjects voluntarily participated after providing oral informed consent. This study was approved on 73/29 September 2021 by Transilvania University of Brasov.

### 2.2. Participants

One hundred seventeen female basketball players, aged between 10 and 12 years (U12), participated in this study. The study subjects were divided into two groups: the experimental group (EG), with 60 (51.3%) subjects, and the control group (CG), with 57 subjects (48.7%). The experimental group was made up of junior U12 athletes from the School Sports Club from Sibiu—the women’s basketball section—and the control group was made up of junior U12 basketball players from the “Gladiu” Sports Club from Targu Mures. Inclusion criteria:

Active athletes, having at least 2 years of experience in basketball, having good health, having no injuries during this study and participation in tests; for the experimental group, inclusion criteria include full performance of the experimental program, the technical level of the subjects according to the primary selection criteria of the Romanian Basketball Federation for U12 (mini-basketball), and the players had to have obtained the minimum score in the physical tests specific to U12. The level of technical skills of the subjects must correspond to the training model for U12 of the FRB, which requires the ability to perform the basic technical and tactical actions in basketball in conditions of adversity and game.

### 2.3. Measures

For the present study, we designed and applied 3 motor tests specific to the basketball game to evaluate the jump shots in conditions specific to the basketball game (the jump shots were preceded by different movements or technical actions): the shot-after-step test, the standing shot test and the shot-after-dribbling test. The tests were applied in the same order for both groups of this study in the initial and final testing. The test application conditions were similar (at the same time interval, after a 30 min warm-up, with the same sensors). The parameters evaluated for each motor test with the MyVert sensor were the maximum height of the jump (cm), the average height of the jump (cm) and the power (watts/kg). We also quantified the number of shots scored during each motor test.

**Figure 1:**
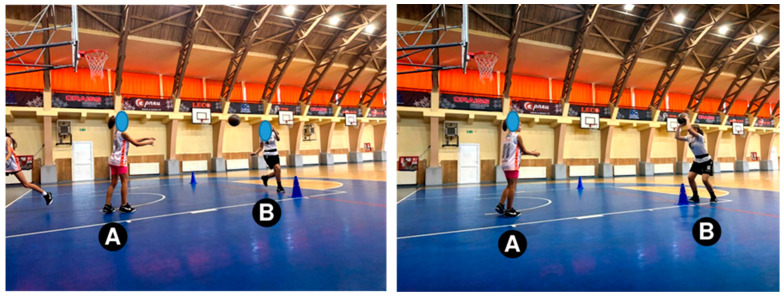
Shot-after-step test.

Shot-after-step test. Starting position: The players are placed as shown in Figure 1. Players denoted with “A” are passers, and player “B” is an executor. Test description: Player “B” executes a run to the marker on the opposite side, receives a pass from player “A” on the same side and executes a 2-time stop and a jump shot, after which she executes the same route back. The route is executed 10 times, and the number of marked shots is noted.

**Figure 2:**
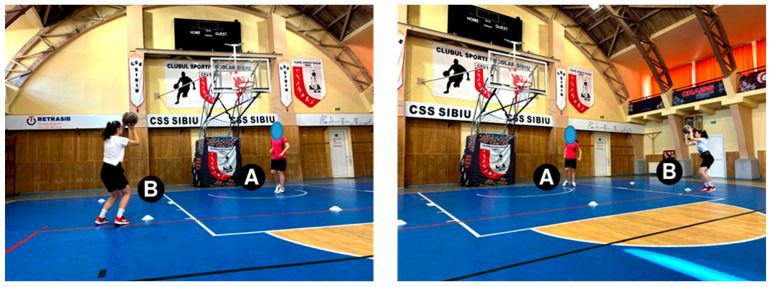
The Standing shot test.

The standing shot test. Initial position: the players are placed as shown in Figure 2. Player “A” is the retriever, and player “B” is the executor. Description of the test: Player “B” performs 2 consecutive shots from each training cone (5 positions), and player “A” recovers her shots and passes the ball back to her. Ten shots are made, and the number of scored shots is noted.

**Figure 3:**
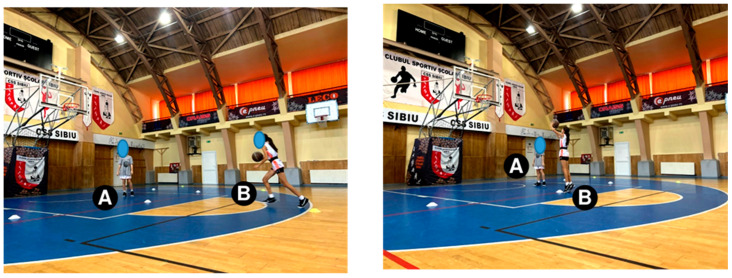
The Shot-after-dribbling test.

The shot-after-dribbling test. Starting position: the players are placed as shown in Figure 3. Player “A” is the passer, and player “B” is the executor. Description of the test: Player “B” dribbles from the training cone (yellow) from the 3-point line, stops in two steps in front of the second training cone (white), jumps, shoots at the basket and returns to the position from the start cone. Player “A” recovers the ball and passes it back to her. Player “B” executes two consecutive shots from each training cone (5 positions). Ten shots were taken at the basket, and the number of scored shots was noted.

### 2.4. Vert Technology Applied in Study

**Figure 4:**
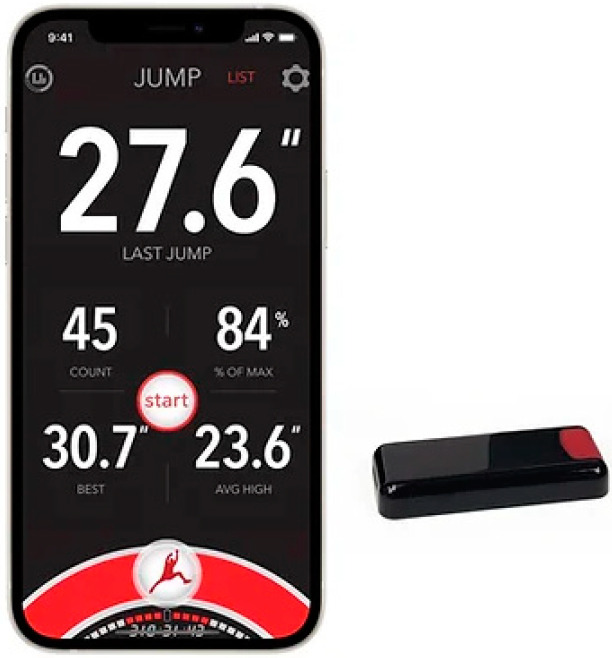
MyVert technology [41].

Vert technology [41] consists of a portable sensor dedicated to sports activity, which is designed for the analysis of vertical jumps by measuring the vertical displacement in proportion to the center of body mass of the subject. The device is made up of a belt where the sensor is placed (the belt is attached to the waist of the athlete) and a mobile application for monitoring the data provided via Bluetooth in the mobile application. Vert sensors include an inertial measurement unit (IMU): 3-axis accelerometer, gyroscope and magnetometer. Vert technology allows real-time monitoring of the following vertical jump parameters: number of jumps, landing impacts, flight height, explosiveness, asymmetry of the athlete, % of max (an athlete’s jump consistency and effort), best (the highest jump during the session and average high (the averaged top 25% of all jumps in a session) (Figure 4), [41]. In our study, we used the Vert sensor in the training process and in the testing of the experimental group; in the control group, it was used only when performing motor tests in order to collect data. The Vert sensors used in this study were used during the experimental program and motor tests. The system has the ability to collect data simultaneously from 16 players. The players in the experimental group were constantly monitored when they performed the exercises specific to the experimental program and during the tests. The collected data allowed the training to be monitored in real time, which facilitated the modeling of the training and the quantification of individual and training group results. In this study, an appropriate number of sensors were used in relation to the size of the group of subjects.

### 2.5. Statistical Analysis

We used IBM-SPSS software, version 22, for statistically processing the following parameters: arithmetic mean (X), standard deviation (SD), mean difference between tests (∆X), Student’s *t*-test (t), confidence interval with lower and upper bounds (95% CI), effect size (d), Skewness and coefficient of variation (CV). Interpretation of Cohen’s d effect size: 0.1–0.2, small; 0.3–0.5, medium; 0.5–0.8, large; and over 0.8, very large. Coefficient of variation interpretation: 0–10% indicates very good homogeneity, 10–20% indicates good homogeneity, 20–30% indicates average homogeneity, and >30% indicates low homogeneity. Fisher’s test, being a statistical hypothesis test, is calculated to highlight whether the variants between two variables are equal. The Skewness parameter was calculated to show the normality of the distribution, which must fall between −1 and 1. We used paired Student’s *t*-test to calculate the differences between the results in the final and the initial testing for the 3 tests of this study of the experimental group, respectively, for the control group. We used independent Student’s *t*-test to highlight the differences between the experimental group and the control group in the initial test, respectively, in the final test and in all 3 tests of this study. Statistical significance was *p* < 0.05 for this study.

## 3. Results

In Table 2, Table 3, Table 4, Table 5, Table 6, Table 7, Table 8, Table 9 and Table 10, we have presented the most relevant results of this study and the relevant statistical parameters in order to highlight the statistical significance and the progress made.

Analyzing the results of the two tests, we find that in the final test, the results of the arithmetic averages show improvements in all the parameters of the shot-after-step test, both for the experimental group and for the control group. The Skewness values were between −0.5 and 0.5, which reflects an almost symmetrical distribution of the results in this test for both study groups in both tests. For the experimental group, during the initial testing, it was found that the CV value was 21.06% for the scored shots, showing a low homogeneity of the group; for the other parameters, the values fell between 10 and 20%, reflecting an average homogeneity. In the final tests of the experimental group, the CV values decreased, reflecting a very good homogeneity (between 1 and 10%) for the jump height and the average height; homogeneity was good for power and scored shots (values between 10 and 20%) (Table 2). For the control group, the CV values in the initial and final testing were higher than those of the experimental group for all parameters of the shot-after-step test, indicating a good and average homogeneity.

**Figure 5:**
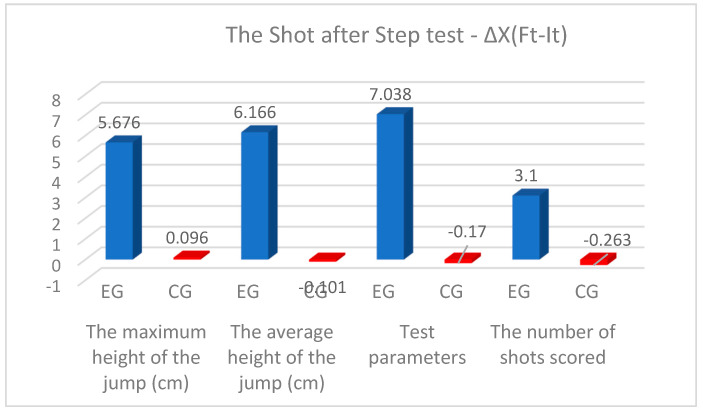
Progress made on the standing shot test (blue color—results of EG; red color—results of CG).

In Table 3, Figure 5, it can be seen that the differences between Ft and It for all the parameters of the shot-after-step test for the experimental group were higher than for the control group. For all test parameters for both groups of this study, the differences between the arithmetic means of the final and initial test fell between the upper and lower limits of the 95% CI. The progress recorded between Ft and It was statistically significant for *p* < 0.05 for all test parameters for the experimental group; for the control group, the differences were statistically insignificant (*p* > 0.05). Analyzing Cohen’s values, we find that for the experimental group, it fell between 1.86 and 3.62, which reflects a very large effect size. For the control group, the effect size was small, with the values falling between 0.02 and 0.33. It should be noted that the greatest progress among the tests was recorded by the experimental group at a power of 7.038 watts/kg and an average height of the jump of 6.166 cm, and the lowest progress was found in scored shots with 3.100 throws. In the control group, the progress between the tests was recorded only in the maximum height of the jump by 0.096 cm (Table 2). It should be noted that the greatest progress recorded for scored shots was found in the experimental group after 3.100 throws as a result of practicing in the experimental program, while the control group result was 0.263 throws.

Comparatively analyzing the results in Table 4, we find that the differences in the arithmetic averages of the shot-after-step test between the experimental and control groups in the initial tests were statistically significant (Student’s *t*-test) only for the parameters of strength and scored shots where *p* < 0.05; in the final tests, *p* = 0.00 for all the parameters of the shot-after-step test, with the differences being statistically significant. The arithmetic mean differences between the experimental and control groups in the initial and final tests fell between the upper and lower limits of the 95% CI. Analyzing the differences of the arithmetic averages (ΔX) between the two groups of this study, it is found that in the final testing, these differences were much greater than in the initial testing for all the parameters of the shot-after-step test, which was in favor of the experimental group. Analyzing the number of shots scored in this test, we find that in the initial test, the experimental group scored 0.555 fewer shots on average than the control group, but in the final test, the progress of the experimental group compared to the control group was 2.806 shots.

In the standing shot test, we find that for the experimental group, the average results from the final test are better than those from the initial test; in the control group, the same situation is found with a single exception, the power, where in the final test the result was lower than in the initial one. The initial arithmetic mean values for all tests for both groups of this study were lower than for the final tests, which highlights that the experiment program produced positive effects. For the experimental group, the CV values fell between 12.45% and 25.97% in the initial test, which reflects a good and average homogeneity of the group, and in the final test, the homogeneity improved, falling between 7.94% and 18. 88%. For the control group, the CV values in the initial and final testing were higher than those of the experimental group for all parameters of the standing shot test, indicating good and average homogeneity. Most of the Skewness values were between −0.5 and 0.5, with a single exception for the power at EG in the initial testing, which reflects an almost symmetrical distribution of the results at this test for both study groups at both tests (Table 5).

**Figure 6:**
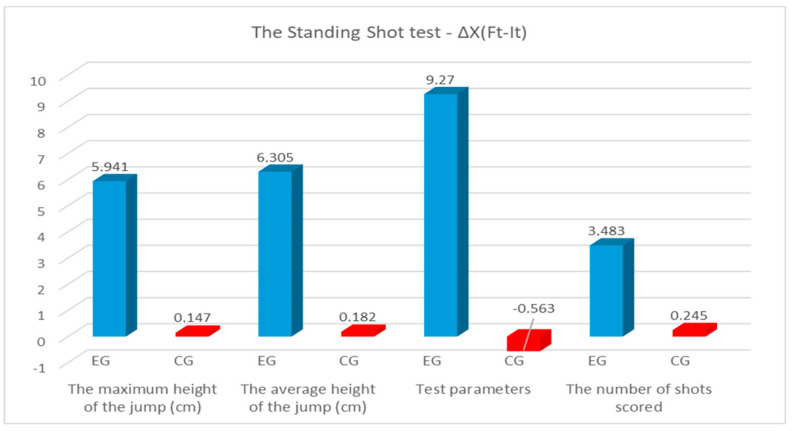
Progress made on the standing shot test (blue color—results of EG; red color—results of CG).

According to Table 6, Figure 6, in the standing shot test, we identified progress in all parameters between the initial and final testing in both groups, but those of the experimental group were higher than those of the control group. The progress recorded between Ft and It was statistically significant for *p* < 0.05, for all test parameters, only in the experimental group. For all test parameters for both groups of this study, the differences between the arithmetic means of the final and initial test fell between the upper and lower limits of the 95% CI; the results show that they can be considered reliable for this study. Analyzing Cohen’s values, we find that for the experimental group, it fell between 1.98 and 4.27, which reflects a very large effect size and emphasizes that the practiced experimental program was effectively reflected by the great progress made between initial and final tests. For the control group, the effect size was small, with the values falling between 0.04 and 0.55. It should be noted that the greatest progress among the tests was registered by the experimental group with a power of 9.270 watts/kg and at the average height of a jump of 6.305 cm, and the lowest progress was in scored shots with 3.483 throws. In the control group, progress between the tests showed that large progress was recorded in scored shots of 0.245 and with the average height of the jump of 0.182 cm, and in strength, the difference was negative (Table 6). The progress of the experimental group was much higher than the control group in all parameters of the standing shot test, which demonstrates the positive impact of the training program implemented through the use of Vert technology. This aspect is especially noticeable in scored shots, where the experimental group progressed with 3.483 shots, while the control group progressed with only 0.245 throws.

Analyzing the results of scored shots, we identify that in the final test, the results of the experimental group were much better, with a score of 2.975, which was more than that of the control group, although, in the initial test, the experimental group had an average result lower than the experimental group with −0.262 shots. In Table 7, it can be seen that the differences between the experimental group and the control group are not statistically significant for the initial testing, with a single exception for power (*p* = 0.00). The arithmetic mean differences between the experimental and control groups in the initial and final tests fell between the upper and lower limits of the 95% CI. The biggest average differences between the study groups were found in the final test at a power of 8.006 watts/kg and an average height of 6.548 cm. For the final testing, the differences between the two study groups are statistically significant for *p* <0.05 in all parameters of the standing shot test.

In the shot-after-dribbling test, the comparative analysis of the results between the two initial and final tests highlights that, in the final test, the results of the arithmetic averages have improvements in all parameters for both groups. The Skewness values reflect an almost symmetrical distribution of the results only for the parameters of the maximum height of the jump, the average height of the jump and scored shots for both groups. Homogeneity of both groups, in both initial and final tests, was good or average for most parameters of the shot-after-dribbling test; we identified a few exceptions where the homogeneity of the experimental group in the final test was very good, with the values being <10% at the maximum height of the jump of 7.54% and at the strength of 7.71% (Table 8).

**Figure 7:**
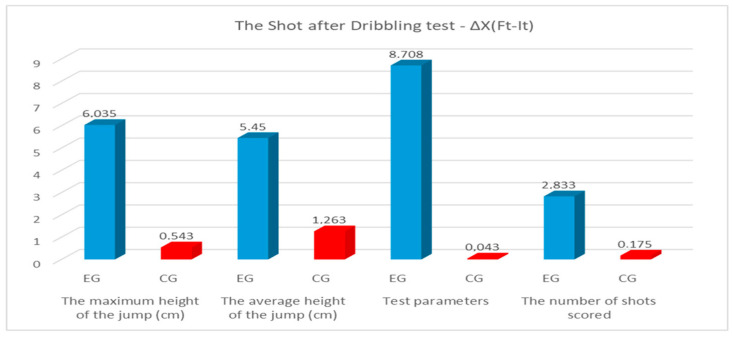
Progress made on the shot-after-dribbling test (blue color—results of EG; red color—results of CG).

According to Table 8, Figure 7, we find that in the shot-after-dribbling test of the experimental group, Cohen’s values fell between 1.66 and 5.88, which reflects a very large effect size. For the control group, the effect size was small, with the values falling between 0.02 for strength and 0.40 for average height. The differences between Ft and It for all parameters of the shot-after-dribbling test for the experimental group were higher than for the control group. For all test parameters for both groups of this study, the differences between the arithmetic means of the final and initial test fell between the upper and lower limits of the 95% CI. The progress recorded between the final and initial testing was statistically significant for *p* < 0.05 for all analyzed parameters in the experimental group, and for the control group, it was only statistically significant for the average jump height. The greatest progress among the tests was recorded by the experimental group at a strength of 8.708 watts/kg and a maximum height of the jump of 6.035 cm, and in the control group, the greatest progress among the tests was recorded at an average height of the jump of 1.263 cm and a maximum height of the jump of 0.543 cm. Analyzing the scored shots, we notice that the experimental group progressed as a result of the implemented experimental program with 2.833 shots, while the control group had only 0.175 shots.

In Table 10, we presented a comparative statistical analysis between the experimental group and the control group in the shot-after-dribbling test in which the differences in the performance of the test parameters of the two groups are highlighted, with a special emphasis on the improvements recorded by the experimental group. According to the Student’s *t*-test, we found that in the final tests, the results between the two groups of this study were not statistically significant, with only one exception in strength; but in the final test, the differences between the experimental and control groups were statistically significant for all the parameters of the shot-after-dribbling test for *p* < 0.05. The arithmetic mean differences between the experimental and control groups in the initial and final tests fell between the upper and lower limits of the 95% CI. According to Table 9, the biggest differences between the study groups in the final test were recorded at the average height of the jump of 7.422 cm and at a strength of 6.914 watts/kg in favor of the experimental group. Analyzing the scored shots, we notice that in the final test, the experimental group progressed with 3.014 throws more than the control group compared to the results from the initial testing, where a small difference of 0.006 shots was recorded, with the groups performing almost similarly (Table 10).

## 4. Discussion

In the present study, we aimed to investigate the impact of the implementation of a 6-month experimental program of combined plyometric and coordination exercises on the basketball jump shot characteristics in conditions close to those of the game in U12 players (girls, juniors). The results of this study highlight the superior progress of the experimental group compared to the control group in all three motor tests specific to basketball. The superior progress recorded by the experimental group, compared to the control group, is considered to be due to the impact of the experimental program implemented in the study of the experimental group. The experimental program applied to the experimental group included exercises that combined coordination exercises with plyometric exercises in the execution of throwing skills and skills specific to the basketball game by using the Vert portable smart sensor (for the entire duration of the experiment). During this study, the control group performed specialized training in which the physical and technical training aimed especially at basketball shots were approached separately through exercises specific to basketball without the use of information technology. The performances of the experimental group improved significantly statistically in terms of the height of the jumps in the execution of the throws (maximum height, average height and strength of the jumps), as well as the number of shots scored in the basket.

The results of this study contribute to the understanding of the manner in which the application of a specialized and adapted workout that combines coordination exercises and plyometrics through the use of intelligent technologies in physical and technical training contributes to the optimization of sports performance in basketball. The relevant results of our study complement previous studies that focused on improving the efficiency of jump shots in training or game conditions through the use of smart technologies and sensors [42,43]. The results of our study align with previous studies that concluded that the use of intelligent technologies that provide real-time data can improve basketball training methodologies based on the information received by coaches and athletes [4,5,19]. Numerous studies have addressed how jumping and basketball shooting can be optimized by specializing and adapting physical and technical training through the use of information technologies that monitor technical and physical parameters in training conditions or during games [44,45,46,47]. A series of studies have evaluated the duration of the jump in making basketball shots [48,49,50,51,52,53,54]. Other studies have focused on the height of the jump, as in the case of our study [48,49,50]. A series of studies carried out on junior basketball players focused on how different technologies contribute to monitoring the parameters of throws in order to adapt training to individual characteristics [51,52,53]. Also, the studies focused on how the efficiency of jump shots is evaluated through different technologies or sensors in conditions of training or during the game [51,52,53,54].

Our study highlights that the implementation of a program of combined plyometric and coordination exercises adapted to the specifics of the basketball game technique contributes to improving the jumping parameters and increasing the efficiency of basketball shots. Other studies have highlighted the contribution of different training programs specialized exclusively for a certain type of exercise on the jumping shot, such as plyometric-only programs [34,55]; others approach the training tools for the development of coordination specific to basketball separately [24,25,37,56,57]. Studies have highlighted that the training of juniors must address the factors of physical and technical training in interrelation to ensure effective motor learning contexts and the improvement of technical skills [58,59,60]. Combining different types of exercises in technical conditions facilitates increasing the attractiveness of basketball training, especially for junior players [58,59]. The advanced training of young people for the practice of basketball requires an approach that uses all the favoring factors and improves the limiting factors specific to the age and the level of sports training [59,60,61,62,63,64]. Sports training in basketball requires a complex and interdisciplinary approach, and the implementation of smart technologies and sensors can contribute to the provision of real-time data that facilitates the analysis and adaptation of training parameters and athletes’ performance. The current trends in the basketball game require modern approaches in which the technology can be efficiently completed in order to optimize sports performance. The results of this study highlight that sports training, combined with different types of methods and exercises, and contributes to improving the level of physical and technical training of basketball players.

The strengths of this study are as follows: the design and implementation of a combined experiment program of plyometric and coordination exercises in order to improve the execution technique and especially the jump shots in conditions close to those of the game, the design of three motor tests to evaluate the jump shots in conditions close to game conditions, the use of the Vert portable intelligent sensor in the process of preparation and motor evaluation and the extended duration of the implementation of the experimental program.

The limitations of this study include the non-inclusion in this study of male samples, the non-inclusion in this study of categories of athletes other than U12, the use of only Vert sensors without the use of other technologies or sensors, the number of throws or jumps made by the control group during the 6 months was not quantified, the environmental factors in which the training was carried out were not taken into account, and the efficiency of the jump shot in official match conditions after the implementation of the experimental program was not analyzed.

## 5. Conclusions

The implementation of a combined experimental program of plyometric and coordination exercises with the use of the Vert portable sensor determined the improvement of the jump shot parameters. The results of the experimental group showed significant statistical progress between the initial and final testing of the experimental group regarding the maximum height of the jump, the average height of the jump and the strength and efficiency of basketball shots. The results of the experimental group after the implementation of the experimental training program that uses Vert technology were statistically significantly superior to the results of the control group. The practical implications of the present study will contribute to the optimization of the athletes’ training methodology in order to improve the physical and technical levels in relation to the age and level of training particularities. We consider that the improvement of coordination and strength abilities in the conditions of practicing basketball throws determines the improvement of the technique and efficiency of basketball throws specific to basketball. Future research directions will be able to focus on the application of combined experimental programs in order to improve various basketball-specific skills and on the use of various informational technologies and portable intelligent sensors specialized in the process of training and monitoring players and the game of basketball, etc. The experimental program can represent a good practice guide for basketball coaches that can be easily adapted in terms of content and dosage for other age categories of juniors. The results of our study corroborated the content of the training program, which will facilitate the understanding of the effectiveness of the combination of plyometric and coordination exercises in improving the level of strength and technique in making jump shots in specific basketball conditions. Specialists in the training of basketball players will be able to use and adapt the experimental program proposed by us to the particularities of the athletes at the training level and use other informational technologies and smart sensors.

## Figures and Tables

**Table 1:** Distribution by months and weeks of the exercises from the experimental program practiced by the experimental group.

Period	Month 1	Month 2	Month 3	Month 4	Month 5	Month 6
Week 1	P1, P2, P3, P4, P5, C-P8, C-P1, C-P2, C-P3	P9, P10, P11, C7, C8, C9, C-P4, C-P5	P15, P16, P17 P18, C11, C-P13, C-P14	P21, P22, C13, C14, C-P17	P26, P27, C14, C-P19, C-P20	P19, P20, P21, C11, C-P9, C-P10
Week 2	P1, P2, P3, P4, P5, C1, C2, C-P1, C-P2, C-P3	P9, P10, P11, C10, C-P7, C-P8, C-P9	P15, P16, P17, P18, C11, C-P13, C-P14	P21, P22, C13, C14, C15, C-P17	P26, P27, C14, C15, C16, C-P19, C-P20	P24, P25, P29, CP9, C-P13, C-P21, C-P22
Week 3	P6, P7, P8, C1, C2, C3, C4, C-P4, C-P5	P12, P13, P14, C10, C-P10, C-P11, C-P7, C-P12	P19, P20, C12, C-P15, C-P16	P23, P4, P25, C15, C16, C-P18	P28, P29, C-P19, C-P20, C-P21, C-P22	P19, P20, P21, C11, C-P9, C-P10
Week 3	P6, P7, P8, C3, C4, C5, C6, C7, C-P5, C-P6	P12, P13, P14, C10, C-P10, C-P11, C-P12	P19, P20, C12, C-P15, C-P16	P23, P24, P25, C16, C-P18	P28, P29, C-P20, C-P21, C-P22	P24, P25, P29, CP9, C-P13, C-P21, C-P22

C—coordination exercises; P—plyometric exercises; C-P—combined exercises.

**Table 2:** Descriptive statistics of the results of the shot-after-step test.

Test Parameters	Group	Test	Min.	Max.	X	SD	Skewness	CV (%)
The maximum height of the jump (cm)	EG	It	21.20	32.30	26.303	3.187	0.020	12.12
		Ft	26.20	36.80	31.980	2.907	−0.052	9.09
	CG	It	18.10	34.00	25.931	4.031	−0.094	15.55
		Ft	19.70	33.70	26.028	2.965	0.013	11.39
The average height of the jump (cm)	EG	It	19.30	31.40	24.666	3.350	0.363	13.58
		Ft	25.60	35.60	30.833	2.900	0.035	9.41
	CG	It	17.10	33.30	24.212	3.941	−0.084	16.28
		Ft	18.40	31.10	24.110	2.727	0.254	11.31
The power (watts/kg)	EG	It	5.40	11.20	7.988	1.494	0.196	18.70
		Ft	10.30	19.70	15.026	2.576	−0.313	17.14
	CG	It	4.10	14.50	9.096	2.548	0.152	28.01
		Ft	5.90	13.70	8.926	2.035	0.553	22.80
The number of shots scored	EG	It	0.00	5.00	2.583	0.544	−0.400	21.06
		Ft	4.00	8.00	5.683	1.081	0.006	19.02
	CG	It	0.00	6.00	3.140	0.821	−0.279	26.15
		Ft	1.00	4.00	2.877	0.746	−0.271	25.93

Min.—minimum; Max.—maximum; SD—standard deviation; CV (%)—coefficient of variation; It—initial test; Ft—final test; EG—experiment group; CG—control group.

**Table 3:** Statistical analysis of the results of the shot-after-step test.

Test Parameters	Group	∆X(_Ft-It_)	SD	95%CI		t	*p*	Effect Size
				Lower	Upper			
The maximum height of the jump (cm)	EG	5.676	1.299	5.340	6.012	33.831	0.000	1.86
	CG	0.096	1.537	−0.311	0.504	0.474	0.638	0.02
The average height of the jump (cm)	EG	6.166	1.842	5.690	6.642	25.919	0.000	1.96
	CG	−0.101	2.317	−0.716	0.513	−0.332	0.741	0.03
The power (watts/kg)	EG	7.038	1.952	6.533	7.542	27.924	0.000	3.34
	CG	−0.170	0.868	−0.400	0.060	−1.479	0.145	0.07
The number of shots scored	EG	3.100	1.068	2.823	3.376	22.466	0.000	3.62
	CG	−0.263	1.587	−0.684	0.157	−1.252	0.216	0.33

∆X(_Ft-It_)—the difference of the arithmetic means; SD—standard deviation; t—value of Student’s test; *p*—statistically significant level.

**Table 4:** Comparative statistical analysis between the experimental group and the control group in the shot-after-step test.

Test Parameters	Test	Fisher’s Test		Student’s *t*-Test		ΔX	95%CI	
		F	*p*	T	*p*		Lower	Upper
The maximum height of the jump (cm)	It (GE-GC)	3.729	0.056	0.555	0.580	0.371	−0.955	1.699
	Ft (GE-GC)	0.008	0.929	10.960	0.000	5.951	4.876	7.027
The average height of the jump (cm)	It (GE-GC)	0.987	0.323	0.673	0.502	0.454	−0.883	1.791
	Ft (GE-GC)	0.390	0.534	12.902	0.000	6.72	5.690	7.754
The power (watts/kg)	It (GE-GC)	17.064	0.000	−2.886	0.005	−1.108	−1.868	−0.347
	Ft (GE-GC)	3.638	0.059	14.163	0.000	6.100	5.247	6.953
The number of shots scored	It (GE-GC)	2.523	0.115	−2.359	0.020	−0.557	−1.024	−0.089
	Ft (GE-GC)	1.296	0.257	14.907	0.000	2.806	2.433	3.179

F—value of Fisher’s test; *p*—value of statistical probability; t—values of independent Student’s test; ΔX—difference of mean averages; CI—interval of confidence; EG—experiment group; CG—control group; It—initial test; Ft—final test.

**Table 5:** Descriptive statistics of the results of the standing shot test.

Test Parameters	Group	Test	Min.	Max.	X	SD	Skewness	CV (%)
The maximum height of the jump (cm)	EG	It	18.00	33.90	26.530	3.304	−0.315	12.45
		Ft	26.80	36.60	32.471	2.641	−0.199	8.13
	CG	It	20.70	31.70	26.582	2.736	−0.365	10.29
		Ft	19.00	35.40	26.729	3.959	−0.055	14.81
The average height of the jump (cm)	EG	It	17.30	33.90	25.415	3.268	−0.192	12.86
		Ft	26.10	35.80	31.720	2.518	−0.204	7.94
	CG	It	18.40	34.60	24.989	4.030	0.198	16.13
		Ft	18.20	30.00	25.171	3.135	−0.466	12.45
The power (watts/kg)	EG	It	6.00	15.10	8.371	2.025	1.449	24.19
		Ft	14.20	20	17.64	1.398	0.408	7.93
	CG	It	5.60	13.90	10.198	2.281	−0.283	22.37
		Ft	6.00	13.00	9.635	1.727	0.077	17.92
The number of shots scored	EG	It	0.00	4.00	1.983	0.515	0.159	25.97
		Ft	4.00	7.00	5.466	1.032	0.092	18.88
	CG	It	0.00	4.00	2.245	0.499	−0.174	22.23
		Ft	0.00	6.00	2.491	0.377	0.656	15.13

Min.—minimum; Max.—maximum; SD—standard deviation; CV (%)—coefficient of variation; It—initial test; Ft—final test; EG—experiment group; CG—control group.

**Table 6:** Statistical analysis of the results of the standing shot test.

Test Parameters	Group	∆X(_Ft-It_)	SD	95%CI		t	*p*	Effect Size
				Lower	Upper			
The maximum height of the jump (cm)	EG	5.941	1.168	5.639	6.243	39.380	0.000	1.98
	CG	0.147	2.122	−0.415	0.710	0.524	0.602	0.04
The average height of the jump (cm)	EG	6.305	1.414	5.939	6.670	34.539	0.000	2.16
	CG	0.182	2.277	−0.421	0.786	0.605	0.548	0.05
The power (watts/kg)	EG	9.270	1.926	8.772	9.767	37.271	0.000	3.79
	CG	−0.563	0.990	−0.826	−0.300	−4.292	0.000	0.27
The number of shots scored	EG	3.483	1.589	3.072	3.893	16.980	0.000	4.27
	CG	0.245	1.628	−0.186	0.677	1.138	0.260	0.55

∆X(_Ft-It_)—the difference of the arithmetic means; SD—standard deviation; t—value of Student’s test; *p*—statistically significant level.

**Table 7:** Comparative statistical analysis between the experimental group and the control group in the standing shot test.

Test Parameters	Test	Fisher’s Test		Student’s *t*-Test		ΔX	95%CI	
		F	*p*	t	*p*		Lower	Upper
The maximum height of the jump (cm)	It (GE-GC)	3.804	0.054	−0.093	0.926	−0.052	−1.166	1.061
	Ft (GE-GC)	8.886	0.004	9.270	0.000	5.741	4.514	6.968
The average height of the jump (cm)	It (GE-GC)	2.608	0.109	0.629	0.531	0.425	−0.915	1.766
	Ft (GE-GC)	2.170	0.143	12.483	0.000	6.548	5.5091	7.587
The power (watts/kg)	It (GE-GC)	1.924	0.168	−4.585	0.000	−1.826	−2.615	−1.037
	Ft (GE-GC)	0.359	0.550	27.612	0.000	8.006	7.432	8.580
The number of shots scored	It (GE-GC)	0.249	0.619	−1.189	0.237	−0.262	−0.699	0.174
	Ft (GE-GC)	3.077	0.082	13.263	0.000	2.975	2.531	3.419

F—value of Fisher’s test; *p*-value of statistical probability; t—values of independent Student’s test; ΔX—difference of mean averages; CI—interval of confidence; EG—experiment group; CG—control group; It—initial test; Ft—final test.

**Table 8:** Descriptive statistics of the results of the experimental group in the shot-after-dribbling test.

Test Parameters	Group	Test	Min.	Max.	X	SD	Skewness	CV (%)
The maximum height of the jump (cm)	EG	It	20.90	32.80	26.585	3.076	−0.268	11.57
		Ft	28.20	36.80	32.631	2.460	0.170	7.54
	CG	It	17.80	32.70	26.243	3.214	−0.308	12.25
		Ft	17.10	31.70	26.787	3.233	−0.565	12.07
The average height of the jump (cm)	EG	It	19.70	31.90	25.323	2.868	−0.085	11.33
		Ft	18.00	35.40	30.773	3.638	−1.424	11.82
	CG	It	17.00	30.10	23.350	3.041	−0.063	13.02
		Ft	16.60	30.60	24.614	3.253	−0.430	13.22
The power (watts/kg)	EG	It	5.40	12.70	8.045	1.645	0.769	20.45
		Ft	14.10	19.30	16.753	1.292	0.160	7.71
	CG	It	6.90	19.70	9.838	2.157	1.896	21.93
		Ft	5.60	18.40	9.882	2.605	1.230	26.36
The number of shots scored	EG	It	0.00	4.00	2.216	0.622	−0.143	28.07
		Ft	4.00	7.00	5.050	0.928	0.293	18.38
	CG	It	0.00	4.00	2.035	0.701	−0.238	34.45
		Ft	0.00	5.00	2.210	0.820	−0.053	37.10

Min.—minimum; Max.—maximum; SD—standard deviation; CV (%)—coefficient of variation; It—initial test; Ft—final test; EG—experiment group; CG—control group.

**Table 9:** Statistical analysis of the results of the shot-after-dribbling test.

Test Parameters	Group	∆X(_Ft-It_)	SD	95%CI		t	*p*	Effect Size
				Lower	Upper			
The maximum height of the jump (cm)	EG	6.035	1.348	5.686	6.383	34.671	0.000	2.17
	CG	0.543	2.372	−1.173	0.085	−1.731	0.089	0.16
The average height of the jump (cm)	EG	5.450	2.916	4.696	6.203	14.476	0.000	1.66
	CG	1.263	2.573	−1.945	−0.580	−3.706	0.000	0.40
Test parameters	EG	8.708	1.325	8.365	9.050	50.893	0.000	5.88
	CG	0.043	1.008	−0.311	0.223	−0.329	0.744	0.02
The number of shots scored	EG	2.833	1.520	2.440	3.226	14.438	0.000	3.58
	CG	0.175	1.881	−0.674	0.323	−0.704	0.484	0.22

∆X(_Ft-It_)—the difference of the arithmetic means; SD—standard deviation; t—value of Student’s test; *p*—statistically significant level.

**Table 10:** Comparative statistical analysis between the experimental group and the control group in the shot-after-dribbling test.

Test Parameters	Test	Fisher’s Test		Student’s *t*-Test		ΔX	95%CI	
		F	*p*	t	*p*		Lower	Upper
The maximum height of the jump (cm)	It (GE-GC)	0.023	0.879	−0.347	0.729	−0.202	−1.358	0.952
	Ft (GE-GC)	2.001	0.160	12.083	0.000	6.376	5.330	7.421
The average height of the jump (cm)	It (GE-GC)	1.337	0.250	1.252	0.213	0.709	−0.412	1.831
	Ft (GE-GC)	0.726	0.396	11.940	0.000	7.422	6.191	8.653
Test parameters	It (GE-GC)	5.384	0.022	−4.585	0.000	−1.837	−2.631	−1.043
	Ft (GE-GC)	4.672	0.033	21.155	0.000	6.914	6.267	7.562
The number of shots scored	It (GE-GC)	0.061	0.805	0.027	0.978	0.006	−0.441	0.453
	Ft (GE-GC)	0.592	0.443	16.039	0.000	3.014	2.642	3.387

F—value of Fisher’s test; *p*—value of statistical probability; t—values of independent Student’s test; ΔX—difference of mean averages; CI—interval of confidence; EG—experiment group; CG—control group; It—initial test; Ft—final test.

## Data Availability

The original contributions presented in this study are included in the article; further inquiries can be directed to the corresponding author.

## References

[1] Papla M., Perenc D., Zając A., Maszczyk A., Krzysztofik M. (2022). Contribution of Strength, Speed and Power Characteristics to Change of Direction Performance in Male Basketball Players. Appl. Sci..

[2] Moanță A.D., Tudor V., Ghițescu I.G. (2013). The methodological overview for the technical-tactical training in basketball. Procedia-Soc. Behav. Sci..

[3] Leidersdorf E., Rauch J., Reeves T., Borkan L., Francis J., Storey L., Souza E.O.D., Elliott M., Ugrinowitsch C. (2022). Reliability and Effectiveness of a Lateral Countermovement Jump for Stratifying Shuffling Performance Amongst Elite Basketball Players. Sports.

[4] Li B., Xu X. (2021). Application of Artificial Intelligence in Basketball Sport. J. Educ. Health Sport.

[5] Benson L.C., Tait T.J., Befus K., Choi J., Hillson C., Stilling C., Grewal S., MacDonald K., Pasanen K., Emery C.A. (2020). Validation of a commercially available inertial measurement unit for recording jump load in youth basketball players. J. Sports Sci..

[6] Nickerson B.S., Medrano N.F., Perez G.L., Narvaez S.V., Carrillo J., Duque M. (2020). Inter-device reliability of wearable technology for quantifying jump height in collegiate athletes. Biol. Sport.

[7] Damji F., MacDonald K., Hunt M.A., Taunton J., Scott A. (2021). Using the VERT wearable device to monitor jumping loads in elite volleyball athletes. PLoS ONE.

[8] Benson L.C., Stilling C., Owoeye O.B.A., Emery C.A. (2021). Evaluating Methods for Imputing Missing Data from Longitudinal Monitoring of Athlete Workload. J. Sports Sci. Med..

[9] Yang Z. (2020). Research on basketball players’ training strategy based on artificial intelligence technology. J. Phys. Conf. Ser..

[10] Hatif B.A., Asrie H.J. (2022). The Effect of Proposed Device on Controlling Shooting Arm Motion’s for Youth Basketball Players. Eur. J. Sports Sci. Technol..

[11] Zhao Y., Xie J. (2017). Artificial intelligence, computer assisted instruction in basketball training. Int. J. Inf..

[12] Yang F., Ren L., Gu C. (2022). A study of college students’ intention to use metaverse technology for basketball learning based on UTAUT2. Heliyon.

[13] Ren L., Yang F., Gu C., Sun J., Liu Y. (2022). A study of factors influencing Chinese college students’ intention of using metaverse technology for basketball learning: Extending the technology acceptance model. Front. Psychol..

[14] Yuan B., Kamruzzaman M.M., Shan S. (2021). Application of Motion Sensor Based on Neural Network in Basketball Technology and Physical Fitness Evaluation System. Wirel. Commun. Mob. Comput..

[15] Yuzhou G., Qi H. (2018). Research on multi direction training and technical analysis of basketball based on BP neural network model. Int. J. Eng. Model..

[16] Petway A.J., Freitas T.T., Calleja-González J., Medina Leal D., Alcaraz P.E. (2020). Training load and match-play demands in basketball based on competition level: A systematic review. PLoS ONE.

[17] Nae C.I., Pop C.L. (2022). Improving university students’ coordinating skills in physical education lessons with basketball focus. Phys. Educ. Stud..

[18] Mohammed N.B., Kzar M.H. (2021). The effect of the use of exploratory exercises in improving concentration of attention and skills of chest handling and correction of basketball stability for people with special needs. Rev. Iberoam. Psicol. Ejerc. Deporte.

[19] Zhao Y., Wang X., Li J., Li W., Sun Z., Jiang M., Zhang W., Wang Z., Chen M., Li W.J. (2023). Using IoT Smart Basketball and Wristband Motion Data to Quantitatively Evaluate Action Indicators for Basketball Shooting. Adv. Intell. Syst..

[20] Zhao B., Liu S. (2021). Basketball shooting technology based on acceleration sensor fusion motion capture technology. EURASIP J. Adv. Signal Process..

[21] Paulauskas R., Balčiūnas M. (2011). Correlation of the Indicators of High Performance Women Basketball Players’ Game Characteristics with Physical Development and Physical Fitness. Balt. J. Sport Health Sci..

[22] Lavrin H. (2017). Technology of concentrated training as one of ways to optimization students’ basketball trainings. Phys. Educ. Stud..

[23] Jerzy S., Paweł W., Janusz Z., Tomasz N., Mariusz B. (2014). Structure of coordination motor abilities in male basketball players at different levels of competition. Pol. J. Sport Tour..

[24] Kamandulis S., Venckūnas T., Masiulis N., Matulaitis K., Balciūnas M., Peters D., Skurvydas A. (2013). Relationship between general and specific coordination in 8- to 17-year-old male basketball players. Percept. Mot. Ski..

[25] Singh H., Saini A. (2017). Relationship of coordinative ability with the skills of basketball. Int. J. Yoga Physiother. Phys. Educ..

[26] Mocanu G.D., Udrea M.G. (2021). The effect of motion games on improving the psychomotor and intellectual performance of children with autism spectrum disorder and intellectual disabilities. Balneo PRM Res. J..

[27] Cojocariu A., Abalasei B. (2014). Does the reaction time to visual stimuli contribute to performance in judo. Arch. Budo.

[28] Demir M.E., Dağlıoğlu Ö. (2022). The effect of plyometric training program on physical performance in basketball players. Eur. J. Phys. Educ. Sport Sci..

[29] Keerthi Kumar M., Urs S.R. (2018). Effect of 12 weeks plyometric training on performance of basketball players. Int. J. Yogic Hum. Mov. Sports Sci..

[30] Ilham I., Muhammad A.M., David I. (2020). The plyometric training on free throw shooting ability and skills in basketball. J. Crit. Rev..

[31] Radenković M., Lazić A., Stanković D., Cvetković M., Đorđić V., Petrović M., Tomović M., Kouidi E., Preljević A., Marković J. (2023). Effects of Combined Plyometric and Shooting Training on the Biomechanical Characteristics during the Made Jump Shot in Young Male Basketball Players. Int. J. Environ. Res. Public Health.

[32] Radenkovic M., Bubanj S., Beric D., Stankovic R., Stojanovic M., Stojic M. (2018). The influence of a ten-week training program on the biomechanical parameters of made jump shots in young basketball players. FU Phys. Educ. Sport.

[33] De Villarreal E.S., Molina J.G., de Castro-Maqueda G., Gutiérrez-Manzanedo J.V. (2021). Effects of plyometric, strength and change of direction training on high-school basketball player’s physical fitness. J. Hum. Kinet..

[34] Selcuk M., Cinar V., Sarikaya M., Oner S., Karaca S. (2018). The effect of 8-week pliometric exercises on some physiological parameters of male basketballers aged 10–14 years. Eur. J. Phys. Educ. Sport Sci..

[35] Cigerci A.E., Genc H. (2020). Plyometric training improves some physical and biomotoric parameters of young male basketball players. Int. J. Appl. Exerc. Physiol..

[36] Chen W.H., Wu H.J., Lo S.L., Chen H., Yang W.W., Huang C.F., Liu C. (2018). Eight-week battle rope training improves multiple physical fitness dimensions and shooting accuracy in collegiate basketball players. J. Strength Cond. Res..

[37] Hadi P., Doewes M., Riyadi S. (2020). The influence of low intensity-high intensity plyometric training and hand-eye coordination on jump shoot ability in basketball players of bhinneka solo club: Randomized control trial. Bp. Int. Res. Crit. Linguist. Educ. J..

[38] Erčulj F., Štrumbelj E. (2015). Basketball shot types and shot success in different levels of competitive basketball. PLoS ONE.

[39] Androutsopoulos P., Blantas I., Papadopoulos K., Lapsanis K., Eleftheriadis G., Alexopoulos P. (2021). The Effectiveness of Plyometric Training in Speed and Agility in Young Basketball Players. J. Mod. Educ. Rev..

[40] Suárez-Iglesias D., Rodríguez-Fernández A., Vaquera A., Villa-Vicente J.G., Rodríguez-Marroyo J.A. (2024). Comparative Effects of Two High-Intensity Intermittent Training Programs on Sub-Elite Male Basketball Referees’ Fitness Levels. Sports.

[41] Vert Technology https://www.myvert.com/.

[42] Abdelrasoul E., Mahmoud I., Stergiou P., Katz L. (2015). The accuracy of a real time sensor in an instrumented basketball. Procedia Eng..

[43] Fan J., Bi S., Wang G., Zhang L., Sun S. (2021). Sensor fusion basketball shooting posture recognition system based on CNN. J. Sens..

[44] Gómez-Carmona C.D., Feu S., Pino-Ortega J., Ibáñez S.J. (2021). Assessment of the Multi-Location External Workload Profile in the Most Common Movements in Basketball. Sensors.

[45] Cheng P.C.Z., Ngali M.Z., Amirnordin S.H., Omar S.F.S., Chin R.Y.S. (2024). Biomechanics Analysis of Normal Jump Shot and Fade-Away Jump Shot in Basketball. J. Adv. Res. Appl. Sci. Eng. Technol..

[46] Rupčić T., Antekolović L. (2016). Application of modern technology in teaching and training with special emphasis on basketball contents. Phys. Educ. New Technol..

[47] Sofyan D., Budiman I.A. (2022). Basketball jump shot technique design for high school athletes: Training method development. J. Sport Area.

[48] Battaglia G., Paoli A., Bellafiore M., Bianco A., Palma A. (2014). Influence of a sport-specific training background on vertical jumping and throwing performance in young female basketball and volleyball players. J. Sports Med. Phys. Fit..

[49] Struzik A., Pietraszewski B., Zawadzki J. (2014). Biomechanical analysis of the jump shot in basketball. J. Hum. Kinet..

[50] Argiriou M., Rousanoglou E., Boudolos K., Bolatoglou T. (2014). The Role of Preceding Technical and Tactical Skills on Jump Shot Accuracy in Male and Female Basketball Players. J. Athl. Enhanc..

[51] Li J., Gu D. (2021). Research on basketball players’ action recognition based on interactive system and machine learning. J. Intell. Fuzzy Syst..

[52] Li L., Wang K. (2022). Research on automatic recognition method of basketball shooting action based on background subtraction method. Int. J. Biom..

[53] Čaušević D., Čović N., Abazović E., Rani B., Manolache G.M., Ciocan C.V., Zaharia G., Alexe D.I. (2023). Predictors of Speed and Agility in Youth Male Basketball Players. Appl. Sci..

[54] Wei W., Qin Z., Yan B., Wang Q. (2022). Application Effect of Motion Capture Technology in Basketball Resistance Training and Shooting Hit Rate in Immersive Virtual Reality Environment. Comput. Intell. Neurosci..

[55] Gül M., Gül K.G., Ataç Ö. (2019). The effect of plyometric trainings on vertical-horizontal jump and some motor skills in U13 basketball players. J. Educ. Train. Stud..

[56] Arede J., Vaz R., Franceschi A., Gonzalo-Skok O., Leite N. (2019). Effects of a combined strength and conditioning training program on physical abilities in adolescent male basketball players. J. Sports Med. Phys. Fit..

[57] Verhoeven F.M., Newell K.M. (2016). Coordination and control of posture and ball release in basketball free-throw shooting. Hum. Mov. Sci..

[58] Gorgan C.M., Oancea B.M., Ciocan C.V. (2024). Using Basketball Game as an Educational Instrument for Childrens Motor Qualities Development. Rev. Rom. Educ. Multidimens..

[59] Gorgan C., Graur C., Jercalau T., Iancu A. (2023). Study regarding the use of dynamic games during the sports training lesson for beginner children, in order to teach them the elements of running. Ann. “Dunarea Jos” Univ. Galati Fascicle XV Phys. Educ. Sport Manag..

[60] Roșu D., Cojanu F., Vișan P.-F., Samarescu N., Ene M.A., Muntean R.-I., Ursu V.E. (2024). Structured Program for Developing the Psychomotor Skills of Institutionalized Children with Special Educational Needs. Children.

[61] Tanasă A.R., Abalașei B.A., Dumitru I.M., Popescu L., Ene-Voiculescu C., Ene-Voiculescu V., Moraru C.E. (2024). Investigating the Influence of Personalized Training on the Optimization of Some Psychomotor Behaviors among Junior Gymnasts in the Training Process (Moldova Region, Romania). BRAIN Broad Res. Artif. Intell. Neurosci..

[62] Badau D., Badau A., Ene-Voiculescu C., Larion A., Ene-Voiculescu V., Mihaila I., Fleancu J.L., Tudor V., Tifrea C., Cotovanu A.S. (2022). The Impact of Implementing an Exergame Program on the Level of Reaction Time Optimization in Handball, Volleyball, and Basketball Players. Int. J. Environ. Res. Public Health.

[63] Badau D., Badau A. (2022). Optimizing Reaction Time in Relation to Manual and Foot Laterality in Children Using the Fitlight Technological Systems. Sensors.

[64] Iconomescu T.M., Mocanu G.D., Talaghir L.G. (2017). The development of conditional motor skills by means of courses and applicative circuits in 6th grade girls during the physical education class. Hum. Sport Med..

